# HIV Testing Practices by Clinical Service before and after Revised Testing Guidelines in a Swiss University Hospital

**DOI:** 10.1371/journal.pone.0039299

**Published:** 2012-06-28

**Authors:** Katharine E. A. Darling, Olivier Hugli, Rachel Mamin, Cristina Cellerai, Sebastien Martenet, Alexandre Berney, Solange Peters, Renaud A. Du Pasquier, Patrick Bodenmann, Matthias Cavassini

**Affiliations:** 1 Infectious Diseases Service, University Hospital of Lausanne, Lausanne, Switzerland; 2 Emergency Department, University Hospital of Lausanne, Lausanne, Switzerland; 3 Service of Immunology and Allergy, University Hospital of Lausanne, Lausanne, Switzerland; 4 Information and Management Control, University Hospital of Lausanne, Lausanne, Switzerland; 5 Service of Psychiatry, University Hospital of Lausanne, Lausanne, Switzerland; 6 Service of Oncology, University Hospital of Lausanne, Lausanne, Switzerland; 7 Service of Neurology, University Hospital of Lausanne, Lausanne, Switzerland; 8 Department of Ambulatory Care and Community Medicine, University of Lausanne, Lausanne, Switzerland; Public Health Agency of Canada, Canada

## Abstract

**Objectives:**

To determine 1) HIV testing practices in a 1400-bed university hospital where local HIV prevalence is 0.4% and 2) the effect on testing practices of national HIV testing guidelines, revised in March 2010, recommending Physician-Initiated Counselling and Testing (PICT).

**Methods:**

Using 2 hospital databases, we determined the number of HIV tests performed by selected clinical services, and the number of patients tested as a percentage of the number seen per service (‘testing rate’). To explore the effect of the revised national guidelines, we examined testing rates for two years pre- and two years post-PICT guideline publication.

**Results:**

Combining the clinical services, 253,178 patients were seen and 9,183 tests were performed (of which 80 tested positive, 0.9%) in the four-year study period. The emergency department (ED) performed the second highest number of tests, but had the lowest testing rates (0.9–1.1%). Of inpatient services, neurology and psychiatry had higher testing rates than internal medicine (19.7% and 9.6% versus 8%, respectively). There was no significant increase in testing rates, either globally or in the majority of the clinical services examined, and no increase in new HIV diagnoses post-PICT recommendations.

**Conclusions:**

Using a simple two-database tool, we observe no global improvement in HIV testing rates in our hospital following new national guidelines but do identify services where testing practices merit improvement. This study may show the limit of PICT strategies based on physician risk assessment, compared to the opt-out approach.

## Introduction

In Switzerland, it is estimated that 18,000 (13,000 - 24,000) adults are living with HIV [1] of whom a substantial proportion are unaware of their status. The rate of late presentation of HIV (defined as patients with CD4 counts <200 cells/µL at diagnosis) remains high, at around 30% [2]. Risk factors for late diagnosis in Switzerland include older age, being married, having children, being heterosexual and, in women, not being pregnant [2]. Late diagnosis is associated with increased morbidity and mortality [3], [4] and increased healthcare costs [5], while HIV-infected individuals unaware of their status do not modify risk behaviour to avoid onward transmission [6].

Having a low threshold for HIV testing is key in identifying patients early in the course of infection. In 2006, the Centers for Disease Control and Prevention (CDC) published revised guidelines on HIV testing which recommend standardised, non-targeted ‘opt-out’ HIV testing for adolescents and adults aged 13 to 64 years in all health care settings, where the prevalence of undiagnosed HIV infection is ≥0.1% [7], [8]. In Switzerland, as elsewhere in Europe, ‘opt-out’ testing has not been adopted. However, in response to accumulating evidence for links between HIV infection and certain risk groups and non-infectious diseases, the Swiss Federal Office of Public Health published revised national testing guidelines in March 2010 recommending Physician-Initiated Counselling and Testing (PICT) [9]. These guidelines maintain the need for prevention counselling and verbal patient consent, and promote testing in four broad clinical settings: 1) symptoms or signs of acute HIV infection (AHI); 2) when HIV enters into the differential diagnoses, such as in sexually transmitted infections, neurological syndromes (dementia, viral meningitis, encephalitis, myelopathy, facial palsy, polyneuropathy) and AIDS-defining illnesses, or when screening is required, as for pregnant women and blood and organ donors; 3) individuals with high risk behaviour and 4) occlusive vascular events (myocardial infarction, stroke, impotence of vascular origin), when testing should be based on risk behaviour assessment [9].

To examine the impact of these revised guidelines on HIV testing practices, we conducted a retrospective analysis of testing practices in different clinical services before (2008–2009) and after (2010–2011) guideline publication. The study took place in our 1400-bed university hospital whose catchment area has an HIV prevalence of 0.4% [1].

## Methods

### Ethics Statement

In accordance with Public Health Legislation of the Canton of Vaud, no ethical approval was required for this study as it was based on two retrospective anonymised databases [10], [11]. Furthermore, our hospital has the authority to employ the General Waiver of the Federal Committee of Experts on Professional Confidentiality in Medical Research and use anonymised data without patient consent and without formal ethical approval, on condition that all patients receive information regarding research activity led by the hospital. The patients may refuse access to their data and this is documented in the administrative system [12], [13].

### Sample Selection

We examined data from the following clinical services: medical outpatients (OP) (comprising three non-specialist clinics: one for minor injuries, one which operates as a public general practice centre by appointment, and one drop-in clinic where no appointment is required); general internal medicine inpatients (IP); neurology IP; cardiology IP (including patients presenting with acute coronary syndromes); intensive care units (ICU); emergency departments (ED, including the acute admission unit); psychiatry IP (excluding services for substance misuse where HIV testing is practised routinely); oncology OP (treating patients with solid organ tumours and lymphoma) and surgery IP (comprising vascular, cardiothoracic, maxillofacial, ear, nose and throat, gastrointestinal, urological and neuro- surgery).

The clinical services were chosen for the following reasons: 1) to examine services dealing with pathologies specifically mentioned in the updated national guidelines (neurological and cardiovascular disease); 2) to examine clinical services recently highlighted in the literature (ICU [14], ED [8], [15] and oncology [16]) and 3) to examine services whose patients have longer hospital stays, a factor previously shown to affect testing practices [15] (internal medicine, psychiatry). All the services examined organise regular intra-service academic and junior staff training activities that are standard in a university hospital; our infectious diseases service provided no specific training in HIV testing as part of this study.

In order to focus on the HIV testing practices in non-specialists in HIV medicine, we did not include the infectious diseases service. We also excluded services where HIV testing is part of specialty protocol: antenatal and reproductive medicine, blood transfusion, transplant medicine and dialysis services (routine HIV screening) and occupational health medicine (screening for HIV and other blood-borne viruses in the context of exposure to biological fluids). To limit our study to testing in adults, as defined by our hospital policy, we excluded individuals aged ≤15 years. Finally, to examine doctor-initiated as opposed to patient-initiated HIV testing, we excluded samples obtained through voluntary anonymous testing.

### HIV Test Requests and Calculation of HIV Testing Rate

All serum samples for HIV testing in our hospital are coded according to the clinical service from which they are sent. HIV testing is performed in the service of immunology and allergy using a fourth generation HIV screening assay which identifies anti-HIV1 and anti-HIV2 antibodies (IgG and IgM) and the p24 antigen (Cobas Elecsys HIV combi PT, Roche Diagnostics). Reactive samples undergo confirmatory tests (neutralisation assay for the p24 antigen (Cobas Elecsys HIV Ag Confirmatory Test, Roche Diagnostics) and a line immunoassay (INNO-LIA™ HIV I/II Score, Innogenetics NV) as well as plasma viral load determination on a second sample (Cobas AmpliPrep/Cobas TaqMan HIV-1, version 2.0, limit of detection at 20 copies/ml, Roche Diagnostics) before they are released as positive for HIV.

Using the service of immunology and allergy database (‘Database 1′), we obtained the number of HIV tests requested by each of the clinical services we wished to examine and the number of samples testing positive. Positive tests were categorised as AHI, new positives (non-acute), known positives (in patients diagnosed previously) and false positives. Only AHI episodes and new positives were used in our analyses. To determine the effect of the revised national HIV testing guidelines published in March 2010, we examined the number of tests performed throughout 2010 and then up to 31^st^ December 2011 to allow for a possible lag period between the guidelines publication and their integration into clinical practice. To compare testing practices pre- and post-guideline publication we collected data from four twelve-month time periods: 1^st^ January to 31^st^ December 2008 and 2009 (pre-guidelines) and 1^st^ January to 31^st^ December 2010 and 2011 (post-guidelines).

In addition to the absolute number of HIV tests requested by each clinical service, we also determined the number of tests performed as a function of the number of patients seen (‘HIV testing rate’), a better measure of testing practices than the absolute number of tests performed that also accounts for any changes in patient turnover observed with time. We obtained figures for patient turnover during the same four twelve-month periods (2008 to 2011) from the central hospital database (‘Database 2’). We examined the number of patients seen, rather than the number of patient episodes, based on the observation that many of the OP services will see a single patient more than once, thereby potentially falsely reducing testing rates in OP compared to IP services.

After ensuring that each patient tested was tested only once in each twelve-month period studied, we expressed HIV testing rate for each clinical service as a percentage using the following equation:

(No. of HIV tests performed in 12-month period/No. of patients seen during the same period) x 100.

Using Database 2, we also obtained figures for median duration of stay (MDOS) among hospital IP, and mean age for all patients.

### Statistical Analyses

Descriptive analyses used means with standard deviation or medians with interquartile range. Categorical data were assessed in two-way contingency table analyses using the Chi squared test. Continuous data were analysed using Student’s t-test and Spearman’s rank correlation coefficient. All statistical analyses were performed using STATA version 11 (Stata Corporation, College Station, TX, USA) and Microsoft Excel 2008 (Microsoft Corporation, Redmond, WA, USA).

## Results

### Patient Turnover

**Figure 1:**
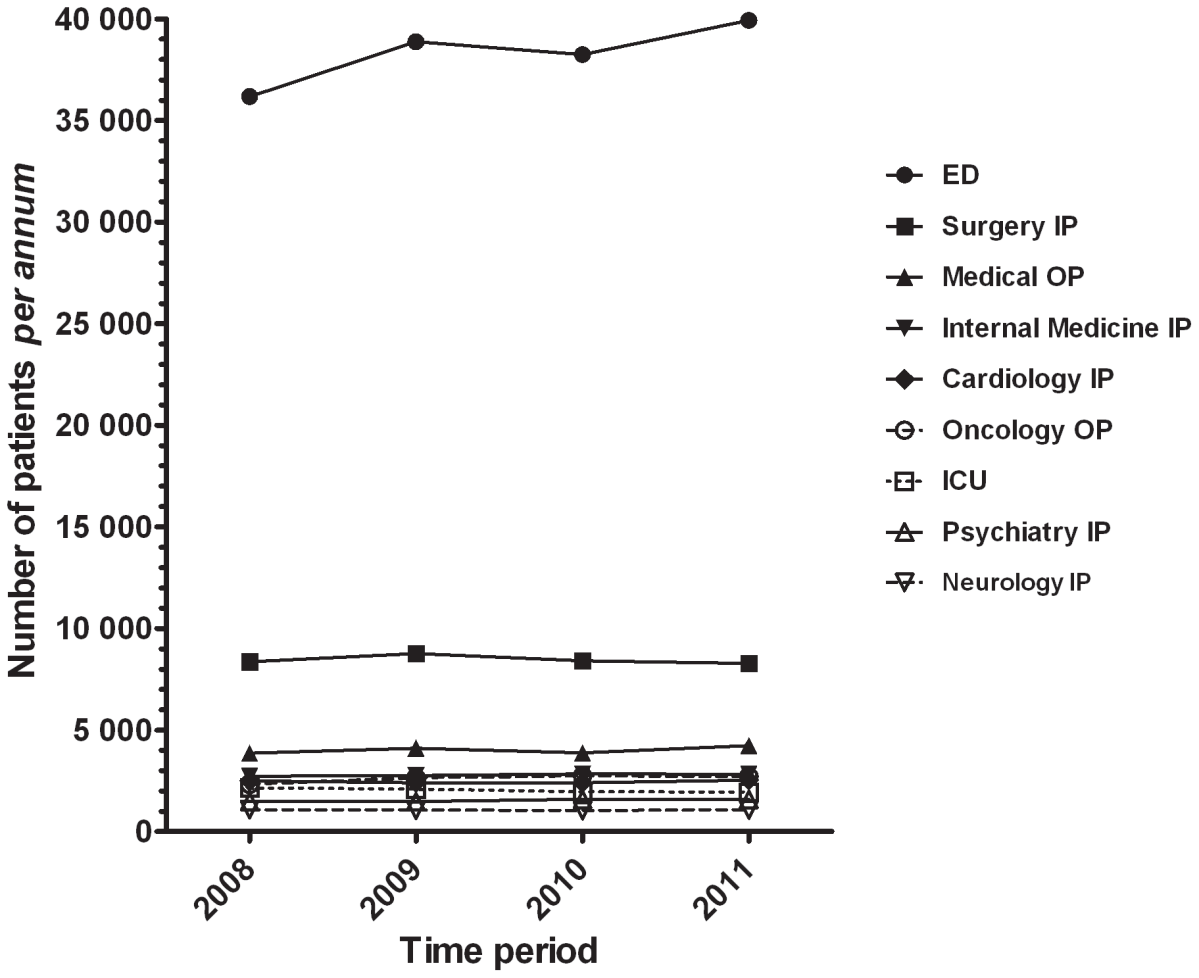
Numbers of patients seen in each clinical service during the four twelve-month periods studied. Abbreviations: OP, outpatients; IP, inpatients; ICU, intensive care units; ED, emergency departments.

Combining the clinical services examined, a total of 253,178 patients were seen between 1^st^ January 2008 and 31^st^ December 2011. By far the majority of these (mean 60.5% of total; SD 0.5%) were seen in the ED (Figure 1). The total number of patients seen increased progressively from 2008 to 2011; increases were observed specifically in the ED, psychiatry, oncology and internal medicine services, whilst numbers in the other services remained static or declined with time (Figure 1).

### Number of Tests Performed per Twelve-month Period

**Figure 2:**
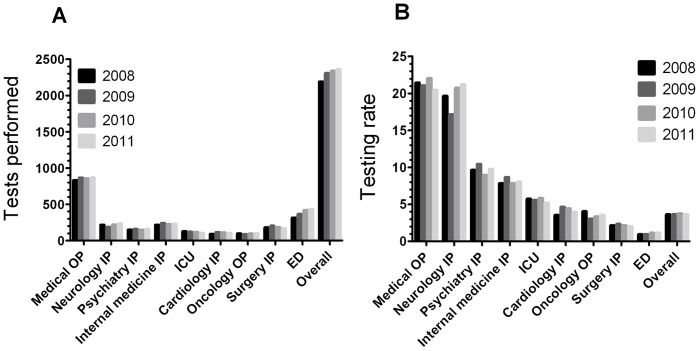
HIV testing practices by clinical service and year. Figure shows the numbers of tests performed (A) and the testing rates (B) in each clinical service during each of the four twelve-month periods studied. Abbreviations: OP, outpatients; IP, inpatients; ICU, intensive care units; ED, emergency departments.

Combining the clinical services, a total of 9,183 tests were performed, with numbers increasing with time (Figure 2A). By far the greatest number of tests were performed in medical OP (Figure 2A), accounting for 36.5–37.8% of all tests performed. After the ED, the other services performed similar numbers of tests, with oncology OP performing the smallest number (Figure 2A). Of all tests performed, 80 were positive (0.9%).

### Number of Tests Performed per Number of Patients Seen (HIV Testing Rate)

Examining HIV testing rates, medical OP had the highest rates, followed by neurology IP (Figure 2B). Although the ED had the second highest number of HIV tests performed, the HIV testing rates were the lowest of all services examined (Figure 2B). Of interest, psychiatry IP had higher testing rates than internal medicine IP, cardiology IP and oncology OP (Figure 2B).

### Effect of Revised National Guidelines on HIV Testing

We compared HIV testing rates from year to year and between the two two-year periods pre- and post-PICT guidelines. Examining all services together, we observed no significant difference in HIV testing rates with time (*P* = 0.94). Examining the subspecialties highlighted in the revised testing guidelines relating to cardiovascular and neurological pathologies, cardiothoracic and vascular surgery had higher testing rates than those of surgery IP as a whole (5% compared to 2.1%, in 2008) but these rates fell in 2010 and 2011; the neurology IP service had high testing rates pre-guidelines (19.7% in 2008) and these remained high but did not increase significantly in 2010 and 2011. Although there was a small increase in testing rates in the cardiology IP service, this was not significant (*P* = 0.7).

In contrast to the other clinical services, the ED testing rates increased from 0.9% to 1.1% between 2008 and 2011. Although the increase from year to year was not significant, the rate throughout 2010 and 2011 (post-PICT guidelines) was significantly higher than that throughout 2008 and 2009 (*P* = 0.011).

### Rate of Samples Testing Positive Among Patients Tested

The percentage of new HIV diagnoses among patients tested for HIV fell from 1.1% (mean; SD 0.2%) pre-PICT guidelines to 0.7% (mean; SD 0.1%) post-guidelines (*P* = 0.3). Of new HIV diagnoses, the percentage of cases of AHI remained static at 8.5–9.4% pre- and post-guidelines. The percentages of positive tests per number of tests performed in each clinical service are shown in Table 1.

**Table 1:** Percentage of tests performed with positive result, median duration of inpatient stay, mean age of all patients seen and mean age of patients tested, for each clinical service.

Clinical service	Percentage of positive tests [%]		Duration of inpatient stay in days[median] (IQR)		Age in years of all patientsseen [mean] (SD)		Age in years of patients tested [mean] (SD)	
	2008–2009	2010–2011	2008–2009	2010–2011	2008–2009	2010–2011	2008–2009	2010–2011
Medical OP	1.2	0.7	N/A	N/A	48.4 (17.1)	48.4 (16.9)	34 (9.4)	34.3 (9.4)
Neurology IP	0.3	0.2	7.4 (5;11.4)	7 (4.4;11.8)	63.1 (19.3)	63.2 (18.8)	49.7 (15.9)	52.5 (16)
Psychiatry IP	0.3	0	18 (8;31)	18 (9;32)	47.5 (19.6)	49 (19.8)	38.9 (11.8)	38.1 (10.6)
Internal medicine IP	0.9	0.5	10.4 (6;16.8)	10 (6;16.9)	71.1 (16.7)	71.4 (16.5)	59.1 (14.8)	59.9 (14.7)
Intensive care units	1.3	0.5	2.1 (1.2;5)	2.4 (1.3;5.3)	61.2 (16.7)	61.2 (16.7)	54.6 (16.5)	57.3 (15)
Cardiology IP	0.5	1.5	3.1 (2;7.6)	3.3 (2;7.9)	64.9 (15)	65.5 (14.8)	54.7 (12.3)	56.7 (12.2)
Oncology OP	0.6	0	N/A	N/A	61.4 (13.2)	61.8 (13)	53.2 (11.6)	53.2 (13.7)
Surgery IP	0.8	0.9	5 (3;10.4)	5.5 (3;11)	57.8 (19.6)	59 (19.8)	55.1 (16.3)	52.5 (14.8)
ED	2.1	1.1	0.7 (0.4;1.1)	1.2 (0.7;2.3)	61.9 (21.1)	62.4 (15.9)	41.2 (13.9)	42.6 (14.3)

As data were not significantly different between 2008–2009 and 2010–2011, they have been pooled for clarity.

Abbreviations: IQR, interquartile range; SD, standard deviation; N/A, not applicable; OP, outpatients; IP, inpatients; ED, emergency department.

### Effect of MDOS and Age on Testing

Of the inpatient services, psychiatry IP had the longest MDOS and ED patients had the shortest (Table 1). Examining all inpatient services together, we did not observe a significant correlation between MDOS and testing rates in either period studied (*P* = 0.27). In all clinical services, the mean age of patients tested was lower than that of all patients seen (Table 1). The difference was most marked in the ED where the mean difference between the ages of patients seen and those tested throughout the study period was of the order of 20 years (Table 1).

## Discussion

Using two hospital databases, we have compared HIV testing rates between different clinical services before and after the publication of revised national testing guidelines. Our data are centre-specific and as such can be interpreted within the framework of our hospital and the organisation of each clinical service.

The relatively high testing rates among neurology IP may be explained by the strong links this service has had with our infectious diseases service since 2006 [17]. Patients with neurological syndromes described in the 2010 guidelines (see introduction) were already being HIV tested by our colleagues prior to 2010, possibly explaining similar testing rates before and after guideline revision. In contrast, the relatively low testing rates among oncology OP are surprising as this service treats lymphoma patients as well as patients with solid-organ cancers. Although solid-organ malignancies are not specifically mentioned in the latest guidelines, there is accumulating evidence that HIV increases the risk of certain such cancers [16], [18]. We can explain the low testing rates by the fact that our hospital is a tertiary referral centre, and that many patients with lymphoma are HIV tested pre-transfer and so would not be tested by our oncology service (S.P., personal communication). However, as one British centre observed that 41% of their lymphoma patients were not HIV tested [19], the testing practices in our oncology service are now part of a collaborative study. Finally, the observation of testing in predominantly younger patients across all clinical services suggests a global belief that HIV is a young person’s disease. This goes against recently reported changes in HIV epidemiology, namely the increasing HIV prevalence in older individuals [4], [20], [21], and suggests that HIV testing competency, even in the better performing services, should be reviewed.

The ED, with the lowest testing rates of all services studied, merit particular consideration. A recent review of ED HIV testing practices placed the median proportion of eligible patients being tested at 22% (range 7–69%) [22]. One study specifically examining the PICT approach observed lower rates at 2.1–2.5% of eligible patients [23]. Of the 0.9–1.1% patients tested in our own ED, we do not have figures on how many patients were ‘eligible’ for testing (see below), but this testing rate, despite the small increase with time, is certainly low compared to the other services we studied. Why are ED testing rates so low? Short duration of stay has previously been cited as an explanation for poor testing rates [15] and certainly the MDOS of patients in our ED was the lowest of all IP services studied. However, that higher testing rates were observed in medical OP compared to all IP services suggests that the length of patient contact is not so critical; perhaps the opportunity for privacy and confidentiality is more important than the time spent with each patient [24]. Perhaps beliefs that HIV risk factors ‘do not relate’ to the primary role of the ED play a role [24], [25]. Whatever the reason(s), as previous studies have shown that measures to optimise ED testing rates produce modest if any improvement [8], [15], we can propose two approaches to optimise testing in patients who pass through this service. The first would be the introduction of rapid testing [26] so that patients can receive their test results in the ED during the shift of the doctor offering the HIV test. The second, more pragmatic, approach would be to focus on improving testing in patients beyond their time in the ED, either in OP follow-up (in our centre, this would be in the medical OP service) or when they are admitted to other IP services.

This study has several limitations. We did not collect data on the presenting complaints of patients tested compared to all the patients seen in each service and so cannot identify testing rates among ‘eligible’ as opposed to all patients seen. Although our testing rates were calculated from patient turnover *per annum*, we did not examine the timing of junior staff turnover, or the use of shift systems within each service, factors which may affect testing practices [25]. That said, we believe these factors to be similar among the acute clinical services. Another potential bias is that of patient origin. Two of the three medical OP clinics we examined receive a high proportion of migrants or political refugees from countries with high HIV prevalence (up to 65% of outpatient episodes, P.B., personal communication), potentially lowering the threshold for HIV testing compared to other clinical services. We do not have figures for migrants attending the other clinical services but, examining country of origin data from Database 2, we can estimate the percentages to be much lower than 65%. Finally, the design of our study does not enable us to identify where the difficulty lies in adopting new guidelines: whether the heads of each service are not aware of them, do not agree with them or have practical difficulties in adhering to them [27]. Determining the barriers to testing will be the subject of further work in the services we have identified with low testing rates.

To summarise, to our knowledge, this is the first study comparing testing rates between different clinical services using the two-database tool we describe. Although our data are centre-specific, our methods for obtaining these data are applicable to any hospital with the appropriate databases. These methods allow under-testing services to be identified and targeted so that missed opportunities for testing, and the associated costs of late HIV diagnosis, may be minimised.
